# Association Between Dietary Diversity and Intrinsic Capacity Among Chinese Older Adults: A Cross-Sectional Study Based on the CLHLS 2018

**DOI:** 10.3390/nu18213568

**Published:** 2026-10-28

**Authors:** Tingting Shi, Mengqing Li, Youyou Zhang, Guanqun Xu, Hui Lu

**Affiliations:** Department of Social Medicine & Implementation Science, Key Laboratory of Public Health Safety and Emergency Prevention and Control Technology of Higher Education Institutions in Jiangsu Province, School of Public Health, Nanjing Medical University, Nanjing 211166, China; shidoubleting@stu.njmu.edu.cn (T.S.); limengqing@stu.njmu.edu.cn (M.L.); 18075474678@163.com (Y.Z.); xgq200265@126.com (G.X.)

**Keywords:** dietary diversity, intrinsic capacity, older adults, healthy ageing, China

## Abstract

**Background:** Dietary diversity has been suggested to play an important role in healthy ageing, yet its association with intrinsic capacity (IC) remains insufficiently explored. This study aimed to examine the association between dietary diversity and intrinsic capacity among older adults. **Methods:** This cross-sectional study included 15,166 participants aged ≥65 years from the 2018 Chinese Longitudinal Healthy Longevity Survey. Dietary diversity score (DDS) was constructed from the reported consumption frequencies of 11 food groups and analyzed both continuously and in quartile groups. IC was constructed as a latent variable using confirmatory factor analysis across five domains: cognition, locomotion, psychological function, sensory function, and vitality. Low IC was defined as a factor score below the sample median. Multivariable regression and restricted cubic spline models assessed associations, supplemented by domain-specific, subgroup, and sensitivity analyses. **Results:** Among 15,166 Chinese adults aged 65 years and older, higher dietary diversity was consistently associated with better intrinsic capacity. In the fully adjusted model, each one-point increase in DDS was associated with a higher IC factor score (*β* = 0.034, 95% CI = 0.029–0.039). Compared with the lowest DDS quartile, the highest quartile had lower odds of low IC (OR = 0.566; 95% CI: 0.493–0.650; *p* for trend < 0.001). Spline analysis supported an inverse association without statistically significant evidence of nonlinearity (*p* = 0.166). Higher DDS was also associated with favorable values across the five domain indicators. Findings persisted after separately excluding the psychological or vitality indicator and using alternative IC thresholds, BMI definitions, and dietary scoring. **Conclusions:** Higher dietary diversity was associated with better IC and lower odds of sample-defined low IC among Chinese older adults. Prospective and intervention studies are needed to clarify the direction of the association and determine whether increasing dietary diversity helps preserve IC.

## 1. Introduction

With the rapid acceleration of global population ageing, achieving healthy ageing has become a primary public health priority [1]. By the end of 2025, China had 323.38 million people aged 60 and above, accounting for 23.0% of the total population, and 223.65 million aged 65 and above (15.9%) [2]. Traditional approaches to ageing research have often emphasized disease status and survival. However, the World Health Organization (WHO) has proposed a function-oriented framework of healthy ageing, defining it as the process of developing and maintaining functional ability in older age [3]. Within this framework, functional ability is determined by the interaction between intrinsic capacity (IC) and the environment. This conceptual shift highlights the importance of identifying modifiable factors that may help preserve functional reserve before the onset of overt disability [4].

IC is defined as the composite of all the physical and mental capacities that an individual can draw upon at any given time [5]. Based on the framework of the International Classification of Functioning, Disability and Health (ICF) and existing evidence, IC is generally categorized into five domains: cognition, locomotion, psychological capacity, sensory capacity, and vitality [6]. These domains encompass cognitive abilities, mobility and muscular function, psychological functioning, vision and hearing, and the physiological processes underlying the maintenance of capacity. They are interrelated rather than independent components of health [6]. Lower IC has been associated with frailty, falls, and other adverse health outcomes [7,8]. By considering several aspects of physical and mental functioning together, IC provides a multidimensional perspective on the health of older adults [9,10].

Although the five-domain framework provides a conceptual basis for assessing IC, the specific operational methods vary across different studies. A systematic review indicated that there are significant differences in the indicators used when evaluating each domain and the methods for obtaining overall scores [11]. The vitality domain faces particular measurement challenges. A working group of WHO defines vitality capacity as a physiological system involving energy and metabolism, neuromuscular function, and immune and stress responses [12]. Therefore, nutritional and anthropometric indicators may only reflect some of the characteristics of this domain and cannot fully represent its complete physiological range.

Nutrition is among the most important modifiable factors for healthy ageing. Older adults are particularly vulnerable to inadequate dietary intake owing to physiological changes, multimorbidity, oral health problems, sensory decline, social isolation, and limited economic resources [13,14,15]. Poor nutrition may contribute to declines in multiple functional domains through reduced muscle mass and strength, impaired immune function, chronic inflammation, oxidative stress, metabolic dysregulation, and neurocognitive changes [13,16,17,18]. Therefore, dietary factors may be closely related not only to individual health outcomes, such as cognition or physical function, but also to the broader construct of IC [19,20].

In recent years, nutritional epidemiology has gradually shifted from the analysis of individual nutrients to the assessment of overall dietary patterns. Dietary diversity describes the variety of foods or food groups consumed. The dietary diversity score (DDS) provides a practical way to summarize reported food-group consumption in population studies [21,22]. Greater dietary diversity may be associated with more adequate nutrient intake, although this relationship also depends on food quantities and combinations [23]. A DDS based on food-consumption frequency therefore does not provide a comprehensive assessment of dietary quality or nutritional adequacy.

Previous studies have linked dietary diversity to cognitive outcomes, depressive symptoms, and broader measures of health in older adults [24,25,26]. Studies have also directly examined dietary diversity in relation to IC [21,27]. However, differences in dietary scoring, domain indicators, and methods of classifying IC complicate comparisons across studies. Further evidence is needed on associations with both overall IC and individual domain indicators in populations with substantial representation of very old adults, and on whether these associations depend on particular exposure and outcome definitions.

This study utilized the data from the 2018 Chinese Longitudinal Healthy Longevity Survey (CLHLS) to explore the association of DDS with continuous IC factor scores and sample-defined low IC among adults aged ≥65 years. The dose–response association with low IC was evaluated, associations with individual domain indicators were examined, and variation across population subgroups was explored. These findings may provide a reference for future research on the relationship between dietary diversity and functional health in later life.

## 2. Materials and Methods

### 2.1. Study Population

This cross-sectional study was based on data from the 2018 wave of the Chinese Longitudinal Healthy Longevity Survey (CLHLS), which was conducted between October 2017 and July 2019. The CLHLS is a national survey covering 23 of the 31 provinces in mainland China and collects information on demographic characteristics, health status, and lifestyle among older adults. The survey was approved by the Biomedical Ethics Committee of Peking University (IRB00001052-13074), and all participants or their legal representatives provided written informed consent. The present study used publicly available, de-identified data and therefore did not require additional ethical approval.

**Figure 1:**
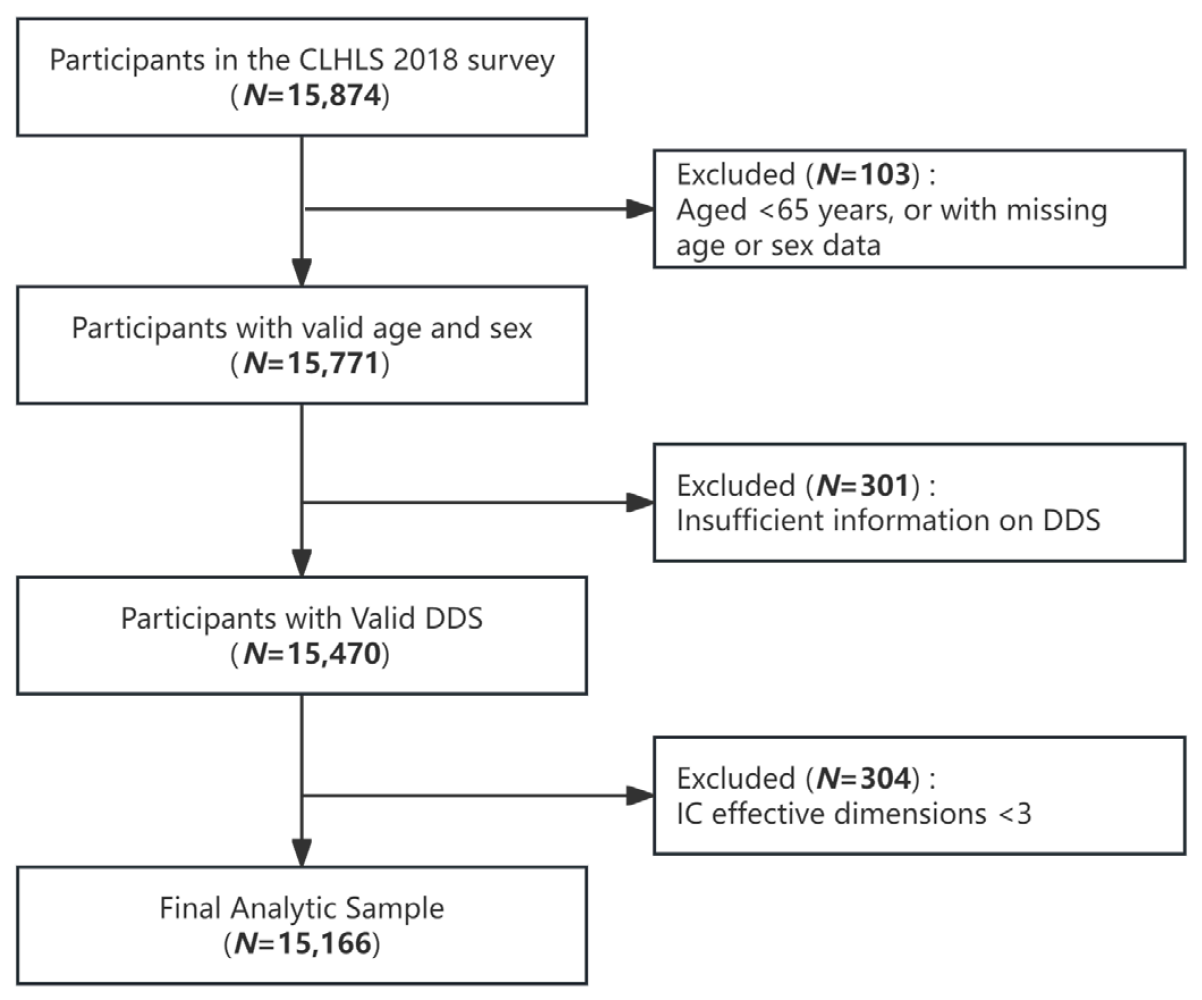
Flowchart of participant selection.

Participants were included if they met the following criteria: (1) aged 65 years or older and non-missing information on key demographic variables, including age and sex; (2) valid responses to at least nine of the 11 food groups required to calculate the dietary diversity score (DDS); (3) valid data for at least three of the five intrinsic capacity (IC) domains. Participants who did not meet these criteria were excluded before multiple imputation. A total of 15,166 participants were included in the final analytic sample. The detailed participant selection process is shown in Figure 1.

### 2.2. Assessment of Dietary Diversity

Dietary diversity was assessed using the Dietary Diversity Score (DDS) based on reported food-consumption frequencies in the CLHLS questionnaire [22]. Of the 13 food categories assessed, sugar and pickled vegetables were excluded from the primary score so that frequent consumption of these items did not increase the score. The primary DDS therefore included 11 food groups: fresh fruits, vegetables, meat, fish, eggs, legumes, dairy products, nuts, algae, garlic, and tea.

For fruits and vegetables, daily consumption was assigned 1 point, and less frequent consumption was assigned 0 points. For the remaining food groups, consumption at least once per week was assigned 1 point, and less frequent consumption was assigned 0 points. Invalid or unknown responses were treated as missing.

For participants with valid responses to at least 9 of the 11 food groups, DDS was calculated as the number of food groups meeting the specified consumption-frequency thresholds divided by the number of food groups with valid responses, multiplied by 11. Scores therefore ranged from 0 to 11. Participants with fewer than nine valid food-group responses were excluded before multiple imputation. Higher scores indicated greater diversity across the assessed food groups.

DDS was analyzed both as a continuous variable and as a categorical variable according to quartiles based on its distribution in the analytic sample. The lowest quartile was used as the reference group in categorical analyses.

### 2.3. Construction and Operationalization of Intrinsic Capacity

IC was operationalized based on the WHO five-domain conceptual framework (cognition, locomotion, psychological capacity, sensory capacity, and vitality) [6]. Given the secondary nature of the CLHLS data, domain indicators were selected according to their conceptual alignment with WHO guidelines, established precedents in published epidemiologic IC research, and data availability [6,11].

(1) Cognition

Cognitive function was assessed using the Mini-Mental State Examination (MMSE), covering orientation, immediate memory, attention and calculation, recall, and language and executive function [28,29]. MMSE scores were calculated when at least 15 items had valid responses. Scores based on available responses were proportionally rescaled to the 0–30 range; participants with fewer than 15 valid item responses were assigned missing scores. The scale demonstrated excellent internal consistency (Cronbach’s α = 0.912). Single-factor confirmatory factor analysis (CFA) supported its construct validity (CFI = 0.925, TLI = 0.920, RMSEA = 0.063, SRMR = 0.068).

(2) Locomotion

Locomotor capacity was evaluated using a 3-item physical performance score (range: 0–6) reflecting daily physical tasks: continuous walking for 2 km, lifting an object weighing approximately 5 kg, and performing repeated squats [6,11]. Each task was scored as 2 (no difficulty), 1 (some difficulty), or 0 (unable to perform). A score was calculated only when all three items had valid responses; otherwise, it was treated as missing. These items capture aspects of mobility and muscular function but do not comprehensively assess locomotor capacity. This domain demonstrated strong internal consistency (Cronbach’s α = 0.909, item-total correlations: 0.806–0.830).

(3) Psychological function

Psychological capacity was represented by depressive symptom burden, capturing the mood-related component of this domain rather than its full scope [30]. Assessment was conducted using the 10-item Center for Epidemiologic Studies Depression Scale (CES-D-10), which includes items concerning depressed mood, fearfulness, loneliness, concentration difficulties, restless sleep, perceived effort, difficulty initiating activities, and positive affect [31].

The mean of the available item scores was calculated when at least seven items had valid responses; otherwise, the composite score was treated as missing. To align its direction with the other IC indicators, the composite score was multiplied by −1, so that higher values indicated fewer depressive symptoms. For the overall IC model, the score was standardized within each imputed dataset before CFA. Internal consistency was good (Cronbach’s α = 0.806). Item-level CFA yielded CFI = 0.958, TLI = 0.936, RMSEA = 0.015, and SRMR = 0.066.

(4) Sensory function

Sensory function was assessed using vision and hearing indicators [6,11,32]. Vision was evaluated using a standard circle-and-gap chart without glasses (2 = correct gap identification, 1 = saw circle only, 0 = blind/cannot see circle). Hearing was assessed via self-reported difficulty (1 = no difficulty, 0 = difficulty). The vision score was divided by 2 to rescale it to a 0–1 range and then averaged with the hearing score using equal weights. The resulting sensory score ranged from 0 to 1, with higher scores indicating better sensory function. The composite score was treated as missing when either component was unavailable. Vision and hearing scores were positively correlated (Spearman’s *r* = 0.310).

(5) Vitality

Vitality encompasses physiological processes related to energy and metabolism, neuromuscular function, and immune and stress responses [12]. BMI was used as an anthropometric proxy for the nutrition-related aspect of this domain [11]. Following the BMI recommendations for older adults issued by the National Health Commission of the People’s Republic of China, age-specific BMI reference ranges were applied: 20.0–26.9 kg/m^2^ for participants aged 65–79 years and 22.0–26.9 kg/m^2^ for those aged ≥80 years [33]. The indicator was coded as 1 for BMI within the specified reference range and 0 otherwise. The indicator was treated as missing when BMI was unavailable. Internal consistency was not assessed for the vitality indicator because it was represented by a single binary measure.

(6) Derivation of Overall IC Factor Score

A one-factor CFA model was fitted to summarize the shared variation among the five domain indicators. Cognition, locomotion, the direction-aligned psychological score, and sensory function were standardized within each imputed dataset, whereas the binary BMI-based indicator was treated as an ordered categorical variable. Models were estimated using weighted least squares with mean- and variance-adjusted test statistics (WLSMV), with the latent factor variance fixed at 1 for identification.

CFA was conducted separately in each of the five imputed datasets. Model fit was evaluated using scaled CFI, TLI, and RMSEA, together with SRMR, and summarized descriptively across imputations. Standardized factor loadings were combined using Rubin’s rules, incorporating the estimate and its sampling variance from each imputed dataset to obtain pooled estimates, standard errors, and 95% confidence intervals. Individual IC factor scores were derived separately within each imputed dataset and oriented so that higher scores indicated better overall capacity.

### 2.4. Covariates

Potential confounders were selected based on the previous literature [34,35], their potential relevance to dietary diversity and intrinsic capacity, and data availability in the CLHLS. These included demographic characteristics, socioeconomic factors, lifestyle factors, and health conditions, as detailed below. All covariates selected for adjustment were retained in the fully adjusted model; none were excluded on the basis of statistical significance, and no stepwise variable selection was performed.

Demographic characteristics included age, sex, residence, and marital status. Age was included as a continuous variable. Sex was categorized as male or female. Residence was categorized as urban or rural. Marital status was categorized as married, separated, divorced, widowed, or never married.

Socioeconomic factors included education level and household income. Education level was categorized as no formal education, primary school, and middle school or above. Household income was assessed as annual household income and was included as a continuous variable.

Lifestyle factors included smoking, alcohol consumption, sleep duration, and regular exercise. Smoking, alcohol consumption and regular exercise were categorized as yes or no. Self-reported sleep duration was recorded in hours and included as a continuous variable.

Health conditions included self-reported physician-diagnosed hypertension, diabetes, arthritis, and dementia, each coded as a binary variable using the corresponding CLHLS questionnaire item.

### 2.5. Statistical Analysis

All statistical analyses were performed using R software (version 4.4.3; R Foundation for Statistical Computing, Vienna, Austria). All tests were two-sided, and a *p*-value < 0.05 was considered statistically significant.

(1) Descriptive Analyses

Continuous variables were summarized as mean ± standard deviation (SD) or median with interquartile range (IQR), as appropriate, and categorical variables were summarized as frequencies and percentages. Percentages were calculated using non-missing observations within each group, and missing observations were reported for each variable. Baseline characteristics were compared across DDS quartiles using analysis of variance, Kruskal–Wallis tests, or chi-square tests, as appropriate. The distribution of DDS was examined using descriptive statistics and histograms.

Among participants aged 65 years or older, characteristics of those included in and excluded from the analytic sample were compared using standardized mean differences to assess potential differences associated with sample selection.

Internal consistency of the cognition, locomotion, and depressive-symptom measures was assessed using Cronbach’s α. The factor structure of the cognition and depressive-symptom measures was examined using item-level confirmatory factor analysis (CFA), with model fit evaluated using CFI, TLI, RMSEA, and SRMR. The correlation between vision and hearing scores was assessed using Spearman’s rank correlation. Internal consistency was not assessed for the BMI-based vitality indicator because it was represented by a single binary measure.

(2) Missing Data and IC Scoring

Before multiple imputation, the proportions of missing values in the final analytic sample were 14.9% for cognition, 0.2% for locomotion, 11.5% for the psychological indicator, 1.3% for sensory function, and 5.9% for the BMI-based vitality indicator.

Missing values in IC indicators and covariates were handled using multiple imputation by chained equations (MICE). Five imputed datasets were generated using 20 iterations. Predictive mean matching was used for continuous variables, logistic regression for binary variables, and multinomial logistic regression for unordered categorical variables. The imputation model included DDS, IC domain indicators, demographic characteristics, socioeconomic factors, lifestyle factors, and health conditions.

CFA models were fitted and IC factor scores were derived separately within each imputed dataset. CFA parameter estimates were combined using Rubin’s rules, whereas fit indices were summarized descriptively across imputations. Low IC was defined within each imputed dataset as an IC factor score below its sample median. This definition represented relatively lower IC within the analytic sample and was not intended as a clinical diagnostic threshold.

(3) Main Association Analyses

Linear regression models examined the association between each one-point increase in DDS and IC factor scores, with results reported as regression coefficients (*β*) and 95% confidence intervals (CIs). Logistic regression models examined associations of continuous DDS and DDS quartile groups with low IC, with results reported as odds ratios (ORs) and 95% CIs. The lowest DDS quartile group was the reference category. Regression models were fitted separately within each imputed dataset, and estimates were combined using Rubin’s rules.

Three models were constructed. Model 1 was unadjusted. Model 2 was adjusted for age, sex, residence, education level, household income, and marital status. Model 3 was further adjusted for smoking, alcohol consumption, sleep duration, regular exercise, hypertension, diabetes, arthritis, and dementia. Tests for linear trend were performed by modeling DDS quartiles as an ordinal variable.

Restricted cubic spline (RCS) regression was used to evaluate the dose–response association between DDS and low IC. Knots were placed at the 5th, 27.5th, 50th, 72.5th, and 95th percentiles of DDS. The RCS model was adjusted for the same covariates as Model 3. The sample median DDS of 5 was the reference value. Overall and nonlinear associations were assessed using pooled multivariate Wald tests with the D1 procedure.

The apparent performance of Model 3 containing DDS quartile groups was described using the area under the receiver operating characteristic curve (AUC) and McFadden’s pseudo-*R*^2^. These measures were calculated separately within each imputed dataset and reported descriptively across imputations.

(4) Domain-Specific and Subgroup Analyses

Domain-specific associations were examined using linear regression for MMSE score, locomotion score, the direction-aligned depressive-symptom score, and sensory function score, and logistic regression for the binary BMI-based vitality indicator. Models were adjusted for the covariates in Model 3, with effect estimates reported per one-point increase in DDS. Because outcomes were measured on different scales, effect estimates were not directly compared across domains.

Subgroup analyses examined associations between continuous DDS and low IC by sex, residence, marital status, education level, smoking status, alcohol consumption, hypertension, diabetes, arthritis, and dementia. Stratified models used the Model 3 covariates, excluding the stratifying variable. Overall multiplicative interactions between DDS and each subgroup variable were assessed using pooled multivariate Wald tests with the D1 procedure. These analyses were exploratory, and interaction findings were interpreted cautiously given multiple comparisons and small sample sizes in some categories.

(5) Sensitivity Analyses

Five sets of sensitivity analyses were conducted to examine whether the findings depended on IC construction, low IC thresholds, or DDS definition. All models used the covariate adjustment specified in Model 3, and regression estimates were pooled across imputed datasets using Rubin’s rules.

First, to assess sensitivity to the inclusion of the BMI-based vitality indicator, IC factor scores were reconstructed after excluding this indicator. Low IC was redefined using the median of the reconstructed scores within each imputed dataset, and associations with DDS quartile groups were re-estimated.

Second, to assess sensitivity to the outcome threshold, low IC was alternatively defined using the lowest tertile and lowest quartile cut-points of IC factor scores within each imputed dataset.

Third, to assess sensitivity to the BMI definition, the vitality indicator was reconstructed using an adult BMI range of 18.5 ≤ BMI < 24.0 kg/m^2^ instead of the age-specific ranges used in the primary analysis. The analytic sample was retained, and multiple imputation, CFA scoring, and median-based low IC classification were repeated.

Fourth, to assess sensitivity to the selection and scoring of food groups, an alternative DDS13 was constructed by incorporating sugar and pickled vegetables. For these two items, consumption less than once per week was assigned 1 point, and consumption at least once per week was assigned 0 points [36]. Scoring of the other 11 food groups was unchanged, and invalid or unknown responses remained missing. At least 10 valid food-group responses were required. Associations using the primary DDS and the alternative reverse-coded DDS13 were compared among participants with valid scores under both definitions. The primary IC classification and imputed covariates were retained.

Fifth, to assess sensitivity to the inclusion of the depressive-symptom indicator, IC factor scores were reconstructed after excluding the psychological domain indicator. Low IC was redefined using the median of the reconstructed scores within each imputed dataset, and the associations between DDS quartile groups and low IC were re-estimated.

## 3. Results

### 3.1. Baseline Characteristics According to DDS Quartiles

The analytic sample included 15,166 participants, with a mean age of 85.39 years (SD 11.56); 43.9% were male. Most participants resided in rural areas, and more than half were widowed. The median annual household income was 20,000 RMB. The proportions of current smokers and alcohol drinkers were 14.9% and 14.3%, respectively. The prevalence of hypertension, diabetes, arthritis, and dementia was 43.1%, 10.2%, 11.6%, and 2.3%, respectively.

Compared with participants in the lowest DDS quartile, those in the highest quartile were younger and more likely to be male, urban residents, married, and educated, and had higher household income. Smoking, alcohol consumption, hypertension, and diabetes also varied across DDS quartiles. Detailed baseline characteristics and missing data are presented in Table 1. A comparison of the characteristics of included and excluded participants is presented in Table S1.

### 3.2. Distribution of DDS and IC Domains

**Figure 2:**
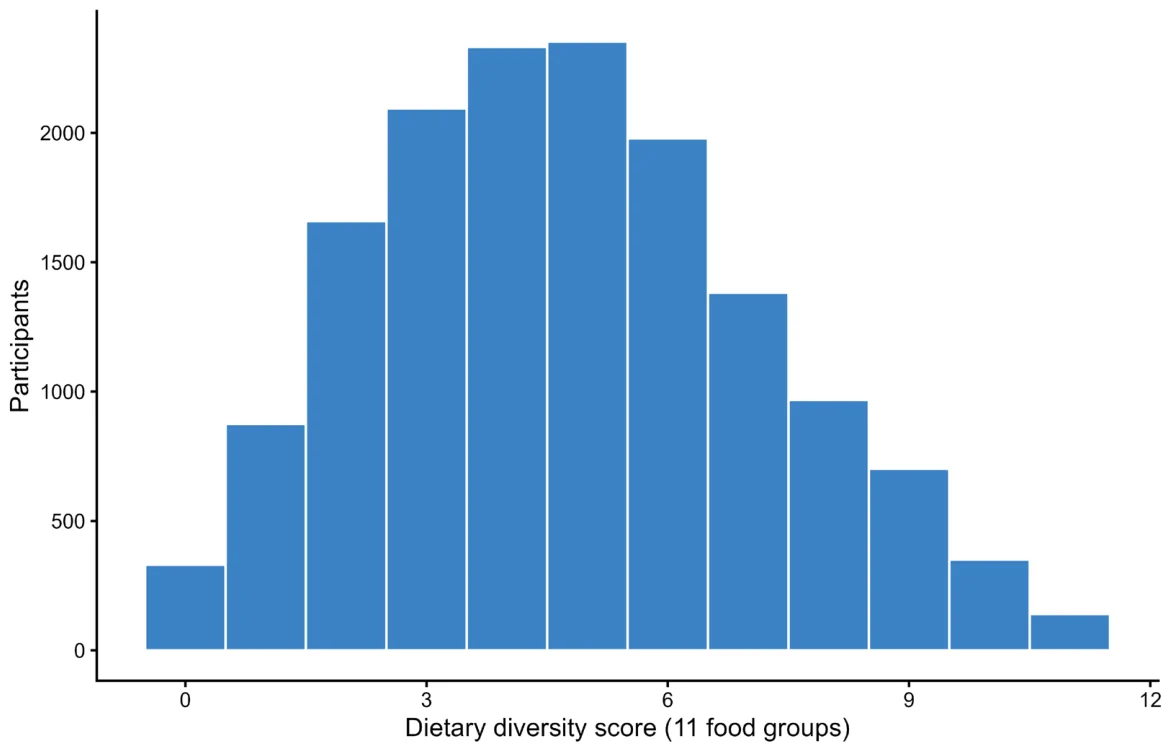
The distribution of DDS.

DDS had a mean of 4.76 (SD 2.41), a median of 5.00 (IQR: 3.00–6.00), and a range of 0–11. Its distribution is shown in Figure 2.

The mean cognition, locomotion, direction-aligned psychological, and sensory scores were 23.45 (SD 8.57), 3.46 (SD 2.46), −0.54 (SD 0.61), and 0.66 (SD 0.36), respectively. Mean BMI was 22.36 (SD 4.20) kg/m^2^. Participants in higher DDS quartile groups had higher mean scores across the four capacity domains. Mean BMI was highest in Q3, followed by Q4. Detailed descriptive statistics are presented in Table 2.

### 3.3. Construction and Assessment of the IC Factor Score

IC was modeled as a latent factor represented by five domain indicators: cognition, locomotion, psychological function, sensory function, and BMI-based vitality. CFA models fitted separately in the five imputed datasets consistently showed good overall fit. Across imputations, CFI ranged from 0.9962 to 0.9974, TLI from 0.9925 to 0.9948, RMSEA from 0.0217 to 0.0262, and SRMR from 0.0107 to 0.0125 (Table 3).

**Figure 3:**
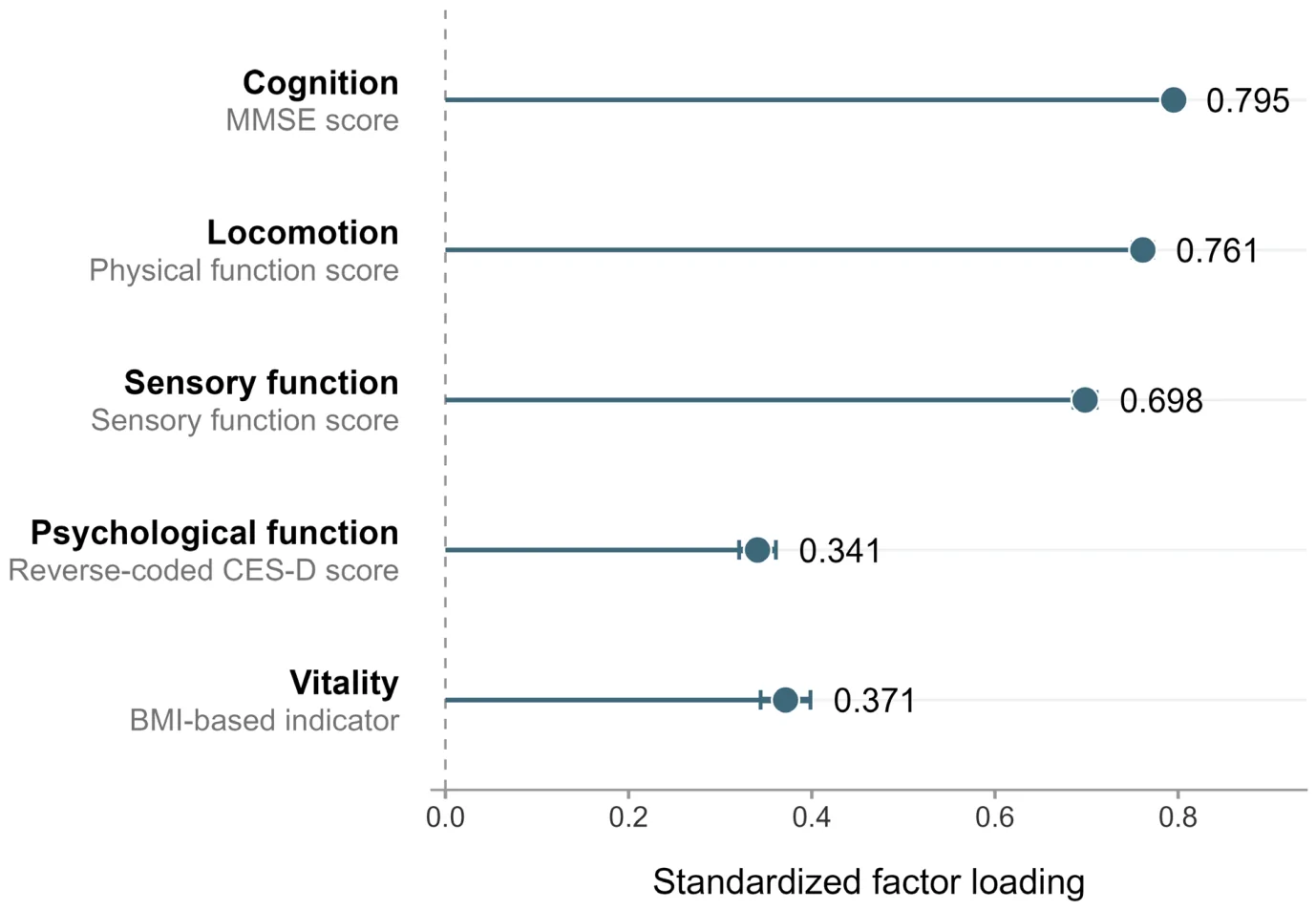
Standardized factor loadings of the five intrinsic capacity indicators. Points represent estimates pooled across five imputed datasets using Rubin’s rules, and short horizontal error bars indicate 95% confidence intervals. The dashed vertical line indicates a loading of zero.

The pooled standardized factor loadings were 0.795 for cognition, 0.761 for locomotion, 0.341 for psychological function, 0.698 for sensory function, and 0.371 for BMI-based vitality (all *p* < 0.001; Figure 3).

### 3.4. Association Between DDS and Overall IC

Table 4 presents the associations of DDS with IC score and low IC. In linear regression analyses, DDS was positively associated with IC scores. After full adjustment for demographic, socioeconomic, lifestyle, and health-related factors, each one-point increase in DDS was associated with a 0.034-unit higher IC factor score (95% CI: 0.029–0.039).

In logistic regression analyses, higher DDS quartiles were associated with progressively lower odds of low IC. Compared with participants in Q1, the fully adjusted ORs for low IC were 0.819 (95% CI: 0.729–0.921) for Q2, 0.672 (95% CI: 0.576–0.784) for Q3, and 0.566 (95% CI: 0.493–0.650) for Q4, with a significant trend across quartiles (*p* for trend < 0.001).

Across the five imputed datasets, the fully adjusted logistic regression model containing DDS quartile groups had a mean apparent AUC of 0.8807 (range: 0.8802–0.8813) and a mean McFadden’s pseudo-*R*^2^ of 0.3733 (range: 0.3721–0.3748). Detailed results are presented in Table S2.

**Figure 4:**
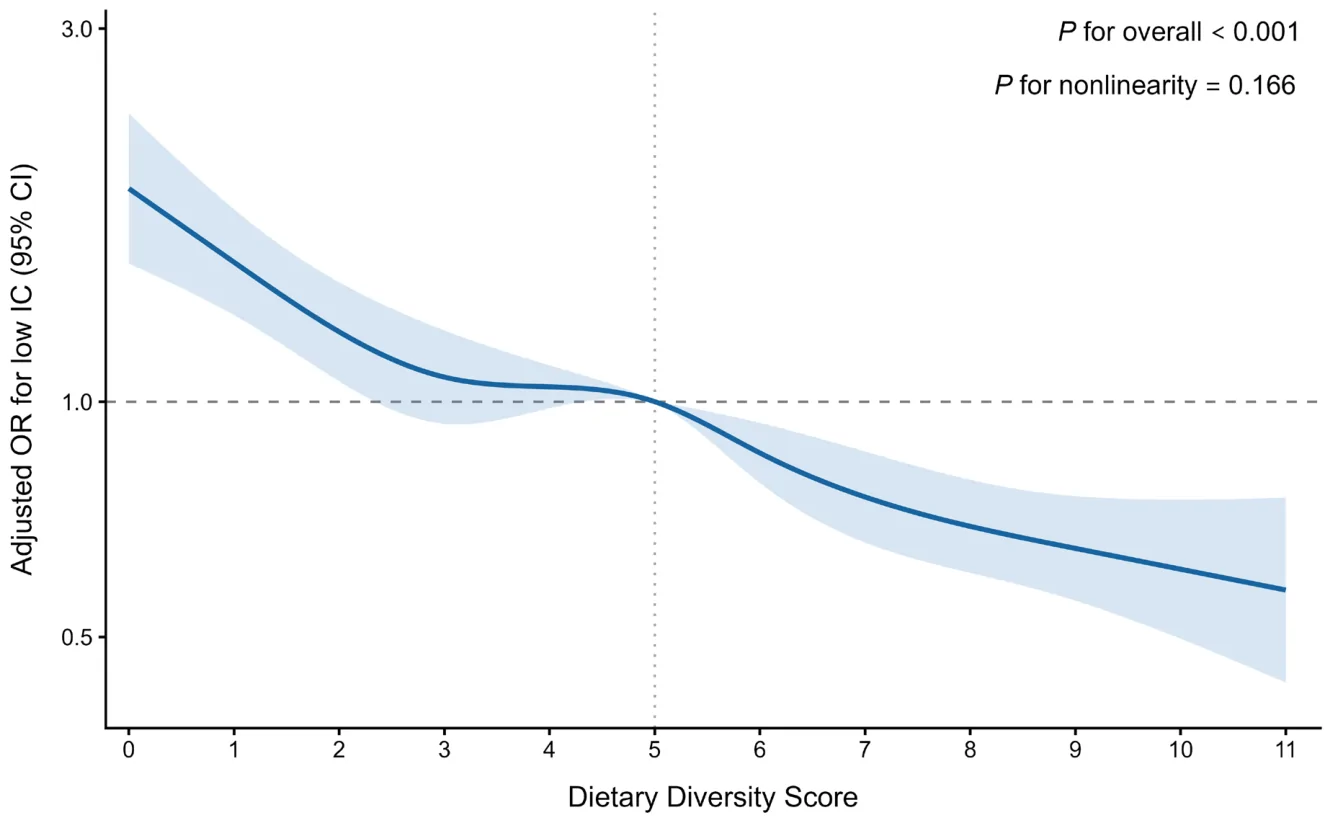
Dose–response relationship between dietary diversity score and low intrinsic capacity. The solid blue curve represents the adjusted odds ratios, and the shaded blue area indicates the 95% confidence intervals. The horizontal dashed line indicates an odds ratio of 1, and the vertical dotted line marks the reference DDS value of 5. The model was adjusted for age, sex, residence, education, income, marital status, smoking, alcohol consumption, sleep duration, regular exercise, hypertension, diabetes, arthritis, and dementia.

Restricted cubic spline analysis showed a significant overall inverse association between DDS and low IC (*p* for overall < 0.001), with no statistically significant evidence of nonlinearity (*p* for nonlinearity = 0.166) (Figure 4).

### 3.5. Domain-Specific Associations

Higher DDS was associated with better cognition, locomotion, and sensory function, and fewer depressive symptoms (Table 5). Each one-point increase in DDS was associated with a 0.305-point higher MMSE score, a 0.052-point higher locomotion score, a 0.050-point higher direction-aligned psychological score, and a 0.008-point higher sensory score (all *p* < 0.001). Higher DDS was also associated with greater odds of having BMI within the specified age-specific reference range (OR = 1.024; 95% CI: 1.007–1.040; *p* = 0.004). Since the indicators are measured on different scales, it is inappropriate to directly compare the magnitudes of the coefficients between different fields.

### 3.6. Subgroup and Sensitivity Analyses

**Figure 5:**
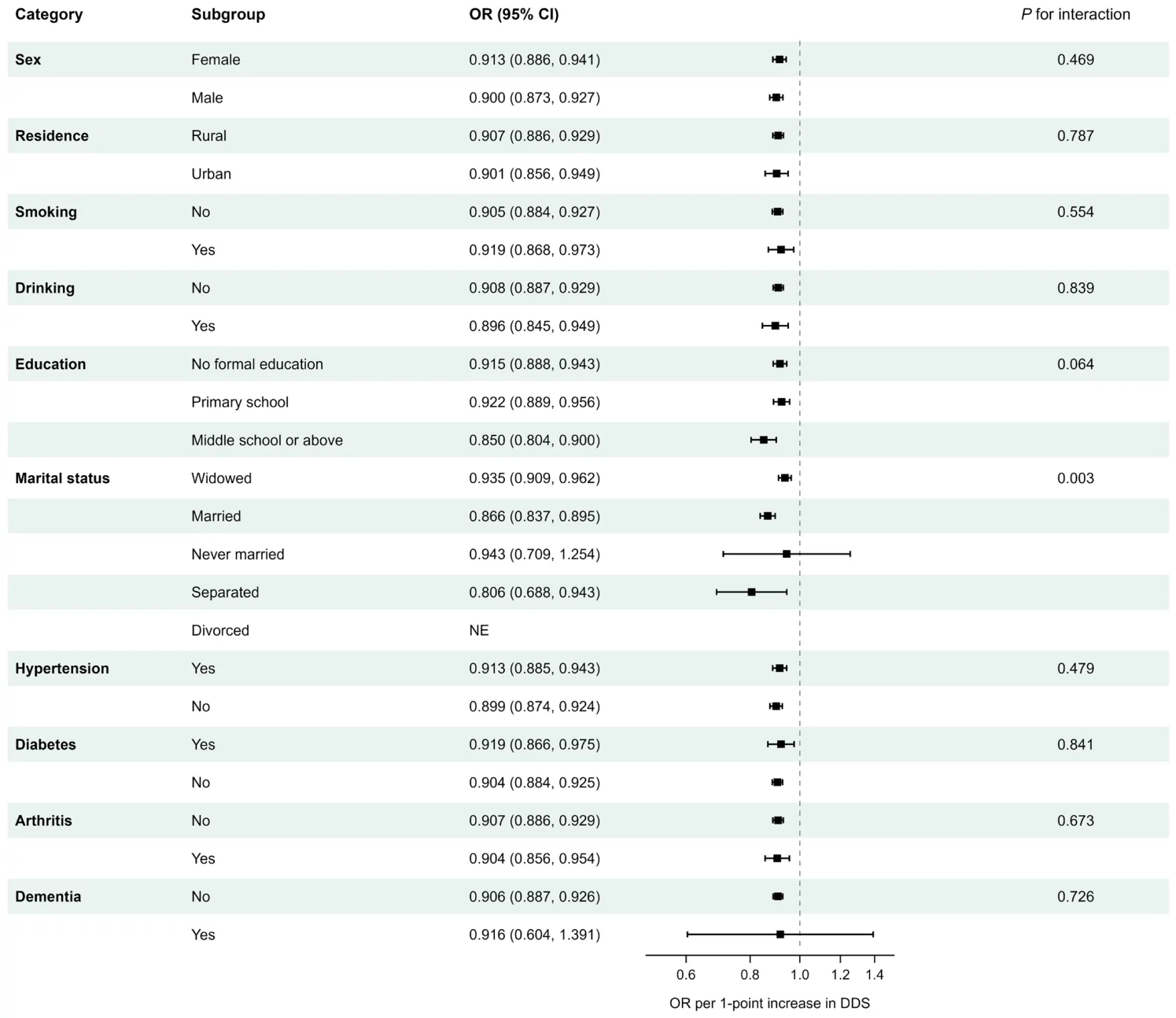
Subgroup analyses of the association between dietary diversity score and low intrinsic capacity. Squares represent adjusted odds ratios, and horizontal error bars indicate 95% confidence intervals. The vertical dashed line indicates an odds ratio of 1. Models were adjusted for age, sex, residence, education, household income, marital status, smoking, alcohol consumption, sleep duration, regular exercise, hypertension, diabetes, arthritis, and dementia, except for the stratifying variable. Odds ratios are expressed per one-point increase in DDS. *p*-values for interaction refer to the overall interaction between DDS and each subgroup variable. NE indicates that an estimate was not available.

The inverse association between DDS and low IC was generally consistent across most subgroups (Figure 5). The overall interaction test for marital status was statistically significant (*p* for interaction = 0.003). Point estimates suggested a stronger inverse association among married than widowed participants, although the omnibus interaction test did not specifically test this pairwise contrast. No statistically significant interactions were observed for the other subgroup variables. These analyses were exploratory and should be interpreted cautiously given the multiple tests and small sample sizes in some categories.

The inverse association between DDS and low IC was consistent across sensitivity analyses (Table S3). Similar associations were observed when IC was reconstructed after separately excluding the BMI-based vitality indicator or the psychological domain indicator, and when low IC was defined using the lowest tertile or quartile of IC scores. The findings also persisted when the vitality indicator was defined using the adult BMI reference range and when dietary diversity was assessed using alternative reverse-coded DDS13.

## 4. Discussion

In this cross-sectional study of 15,166 Chinese older adults, higher DDS was associated with higher overall IC factor scores and lower odds of sample-defined low IC after multivariable adjustment. The association with low IC was generally inverse, without statistically significant evidence of nonlinearity. Higher DDS was also associated with better cognition, locomotion, and sensory function, fewer depressive symptoms, and modestly greater odds of having BMI within the specified age-specific reference range. The overall association persisted across the sensitivity analyses examined.

Within the WHO framework of healthy ageing, IC offers a perspective on physical and mental capabilities to complement the assessment based on diseases [3]. Our findings are broadly consistent with previous studies reporting associations between dietary diversity and cognitive function, depressive symptoms, physical function, and other ageing-related outcomes [37,38,39,40]. Moreover, existing studies have directly explored the relationship between DDS and IC and reported similar correlations. In a cross-sectional analysis of 1972 Chinese older adults from the Fujian Prospective Aging Cohort, higher dietary diversity was associated with greater odds of high IC [21]. A study of 745 community-dwelling older adults in Beijing found that a higher DDS was associated with lower odds of poor IC (adjusted OR per one-point increase: 0.836) [27]. However, differences in food group definitions, dietary scores, domain indicators, and IC classification criteria among various studies have limited direct comparisons between these studies. This study provides new evidence for a large population sample by examining IC factor scores and individual domain indicators and assessing the sensitivity of the research results to different operational definitions.

Although the fitted spline showed local variation in slope, the nonlinear component was not statistically significant (*p* = 0.166). The curve therefore does not establish a DDS threshold or saturation effect.

Several nutritional explanations are plausible. Consuming a wider range of foods may increase opportunities to obtain protein, vitamins, minerals, and other components relevant to physical and mental functioning [18,41,42]. In the PREDIMED-Plus study, lower dietary diversity was associated with a greater likelihood of inadequate nutrient intake [23]. Diet-related interactions with the gut microbiome may also be relevant to healthy ageing [43]. However, the present study did not measure nutrient intake, inflammatory markers, or microbiome characteristics, and cannot determine whether these processes account for the observed association. Dietary diversity may also reflect the resources and abilities needed to obtain, prepare, and consume food. Socioeconomic circumstances, nutritional literacy, and household support have been associated with dietary diversity in older adults [44,45,46]. Such factors could influence both dietary variety and functional health. The association observed here may therefore reflect nutritional pathways as well as shared social and behavioral determinants that were not fully captured by the available covariates.

The domain-specific findings help place the overall association in context. The association with cognition accords with longitudinal evidence linking greater dietary diversity to a lower incidence of cognitive impairment [47]. Similarly, a four-year study of community-dwelling Japanese older adults associated greater dietary variety with lower odds of decline in grip strength and usual gait speed [48]. In the present study, associations with fewer depressive symptoms and better sensory function suggest that the overall finding was accompanied by differences across several measured capacities. These results do not establish which domain was most strongly associated with DDS because the indicators differed in measurement scale, content, and precision.

Interpretation of these findings also requires consideration of how the five domains were represented. The MMSE, physical task items, and vision and hearing measures capture relevant aspects of cognition, locomotion, and sensory capacity, respectively. The psychological indicator represents depressive symptoms rather than the full range of psychological capacity. Although depressive symptoms are considered in the WHO ICOPE assessment approach [49], the instrument and scoring procedure used here differ from those in ICOPE. BMI likewise represents an anthropometric proxy for the nutrition-related aspect of vitality, rather than a direct assessment of energy metabolism or physiological reserve [12]. Good overall CFA fit provides evidence about the relationships among the selected indicators but does not establish complete coverage of each domain. The persistence of the DDS–IC association after separately excluding the psychological or BMI-based indicator suggests that the finding was not attributable solely to either measure.

Subgroup analyses identified an exploratory interaction with marital status. Shared meals and assistance with obtaining or preparing food may be relevant to the observed pattern [50]. Previous studies using CLHLS data demonstrated that older adults cohabiting with a spouse or family members were more likely to maintain higher dietary diversity compared with those living alone [46]. However, given that these subgroups are more exploratory in nature, and that some categories (such as divorced or never married individuals) have relatively small sample sizes, the observed interaction effects of marital status should be interpreted with caution and need to be verified in prospective studies.

The observed association highlights the potential relevance of dietary variety to community services and clinical assessments for older adults. At the policy level, community nutrition programmes could address practical barriers to obtaining and preparing varied foods, including financial constraints, mobility limitations, and limited assistance with shopping or cooking. Nutrition education could be combined with meal services and practical support, particularly for older adults with functional limitations. In clinical practice, questions about regularly consumed food groups could complement nutritional and functional assessments. When restricted dietary variety is identified, further assessment could explore whether it reflects limited food access, difficulties with meal preparation, or a need for assistance with daily activities. Such information may help guide individualized dietary advice and support. However, these implications remain tentative: the present study does not establish that increasing dietary diversity improves IC, and the effects of these approaches require evaluation in prospective and intervention studies.

One advantage of this study lies in the inclusion of a large number of elderly individuals, enabling the exploration of the relationship between dietary diversity and functional ability at the advanced age stage. The examination of continuous IC factor scores alongside domain-specific indicators provided complementary information beyond a binary classification of low IC. Sensitivity analyses further assessed whether the association depended on the inclusion of particular indicators or on the exposure and outcome definitions examined.

Several limitations should be acknowledged. First, the cross-sectional design precludes causal inference and determination of temporal order. Lower capacity may itself restrict dietary variety, and residual confounding by factors such as food access and social support remains possible. Second, IC was operationalized using survey indicators that incompletely represent its domains. The physical task score captures selected aspects of locomotion, the psychological indicator focuses on depressive symptoms, and BMI provides limited information about vitality. Median-defined low IC also represents relatively lower capacity within this sample rather than clinically established impairment. Third, DDS reflects the variety and frequency of selected food groups but does not capture portion sizes, total energy intake, preparation methods, or overall dietary quality. Finally, excluded participants were older, less educated, and more likely to have dementia, raising concerns about selection bias that multiple imputation among included participants cannot eliminate. These differences, together with the advanced age distribution of the analytic sample, warrant caution when generalizing the findings to the broader older population.

## 5. Conclusions

Higher dietary diversity was associated with higher IC factor scores and lower odds of sample-defined low IC in this cross-sectional study of Chinese older adults. The associations were consistent across sensitivity analyses using alternative IC constructions, outcome thresholds, and dietary scoring approaches. Prospective and intervention studies are needed to clarify temporal relationships and determine whether increasing dietary diversity helps preserve intrinsic capacity.

## Figures and Tables

**Table 1:** Baseline Characteristics of Participants.

Characteristics	Overall (*N* = 15,166)	Q1 (*n*_1_ = 4928)	Q2 (*n*_2_ = 4681)	Q3 (*n*_3_ = 2011)	Q4 (*n*_4_ = 3546)	Missing, *n* (%)	*p* Value
Age, years, mean (SD)	85.39 (11.56)	86.92 (11.50)	85.52 (11.64)	85.14 (11.37)	83.24 (11.32)	0 (0.0)	<0.001
Sex, n (%)						0 (0.0)	<0.001
Male	6657 (43.9)	1852 (37.6)	2021 (43.2)	919 (45.7)	1865 (52.6)		
Female	8509 (56.1)	3076 (62.4)	2660 (56.8)	1092 (54.3)	1681 (47.4)		
Sleep duration, hours, mean (SD)	7.45 (2.40)	7.27 (2.55)	7.52 (2.42)	7.56 (2.33)	7.54 (2.20)	631 (4.2)	<0.001
Residence, n (%)						2142 (14.1)	<0.001
Urban	2156 (16.6)	323 (7.9)	472 (11.8)	326 (18.9)	1035 (32.7)		
Rural	10,868 (83.4)	3788 (92.1)	3543 (88.2)	1403 (81.1)	2134 (67.3)		
Marital Status, n (%)						148 (1.0)	<0.001
Married	5962 (39.7)	1635 (33.6)	1764 (38.0)	835 (42.1)	1728 (49.1)		
Separated	266 (1.8)	82 (1.7)	88 (1.9)	33 (1.7)	63 (1.8)		
Divorced	51 (0.3)	14 (0.3)	13 (0.3)	6 (0.3)	18 (0.5)		
Widowed	8613 (57.4)	3094 (63.5)	2732 (58.9)	1092 (55.0)	1695 (48.1)		
Never married	126 (0.8)	46 (0.9)	44 (0.9)	19 (1.0)	17 (0.5)		
Family Income, median [IQR]	20,000 [5000–40,000]	10,000 [4000–30,000]	20,000 [5000–40,000]	20,000 [7000–50,000]	30,000 [10,000–50,000]	4231 (27.9)	<0.001
Education Level, n (%)						2146 (14.2)	<0.001
None	6515 (50.0)	2673 (65.4)	2149 (53.5)	793 (45.7)	900 (28.3)		
Primary	4088 (31.4)	1109 (27.1)	1318 (32.8)	600 (34.5)	1061 (33.3)		
Middle School and above	2417 (18.6)	304 (7.4)	548 (13.6)	344 (19.8)	1221 (38.4)		
Smoking, n (%)						149 (1.0)	<0.001
No	12,778 (85.1)	4250 (86.9)	3898 (84.2)	1675 (84.3)	2955 (84.2)		
Yes	2239 (14.9)	642 (13.1)	733 (15.8)	311 (15.7)	553 (15.8)		
Drinking, n (%)						232 (1.5)	<0.001
No	12,805 (85.7)	4339 (89.2)	3967 (86.1)	1656 (83.7)	2843 (81.7)		
Yes	2129 (14.3)	528 (10.8)	642 (13.9)	323 (16.3)	636 (18.3)		
Regular Exercise, n (%)						209 (1.4)	<0.001
No	10,410 (69.6)	3895 (80.3)	3338 (72.1)	1333 (67.1)	1844 (52.8)		
Yes	4547 (30.4)	953 (19.7)	1291 (27.9)	653 (32.9)	1650 (47.2)		
Hypertension, n (%)						1077 (7.1)	<0.001
No	8023 (56.9)	2681 (60.0)	2621 (59.9)	1057 (56.6)	1664 (49.2)		
Yes	6066 (43.1)	1787 (40.0)	1751 (40.1)	812 (43.4)	1716 (50.8)		
Diabetes, n (%)						1592 (10.5)	<0.001
No	12,189 (89.8)	3990 (93.2)	3865 (90.8)	1598 (88.9)	2736 (84.5)		
Yes	1385 (10.2)	291 (6.8)	392 (9.2)	200 (11.1)	502 (15.5)		
Arthritis, n (%)						1636 (10.8)	<0.001
No	11,961 (88.4)	3808 (89.2)	3785 (88.8)	1604 (89.5)	2764 (86.2)		
Yes	1569 (11.6)	461 (10.8)	475 (11.2)	189 (10.5)	444 (13.8)		
Dementia, n (%)						1671 (11.0)	0.034
No	13,178 (97.7)	4145 (97.3)	4141 (97.6)	1746 (97.5)	3146 (98.3)		
Yes	317 (2.3)	117 (2.7)	100 (2.4)	45 (2.5)	55 (1.7)		

Notes: Data are presented as mean (SD), median [25th–75th percentiles], or *n* (%). Descriptive statistics are based on observed data before multiple imputation in the final analytic sample. Percentages for categorical variables were calculated using non-missing observations within each column and may not sum to 100% because of rounding. The Missing column reports missing observations as a number and percentage of the total analytic sample (*N* = 15,166). Q1–Q4 denote the quartile groups of DDS, from lowest to highest. DDS, Dietary Diversity Score; SD, standard deviation.

**Table 2:** Intrinsic capacity domain scores and BMI according to DDS quartile groups.

Characteristics [Mean (SD)]	Overall (*N* = 15,166)	Q1 (*n*_1_ = 4928)	Q2 (*n*_2_ = 4681)	Q3 (*n*_3_ = 2011)	Q4 (*n*_4_ = 3546)	*p* Value
Cognition Score	23.45 (8.57)	21.59 (9.36)	23.26 (8.50)	24.14 (8.06)	25.84 (7.03)	<0.001
Locomotion score	3.46 (2.46)	3.06 (2.47)	3.45 (2.46)	3.62 (2.44)	3.96 (2.37)	<0.001
Direction-aligned psychological score ^a^	−0.54 (0.61)	−0.69 (0.60)	−0.55 (0.60)	−0.47 (0.60)	−0.36 (0.59)	<0.001
Sensory function Score	0.66 (0.36)	0.60 (0.37)	0.65 (0.37)	0.68 (0.35)	0.73 (0.33)	<0.001
BMI	22.36 (4.20)	22.14 (4.23)	22.17 (4.21)	22.77 (4.12)	22.69 (4.11)	<0.001

^a^ The psychological score is the depressive-symptom score multiplied by −1; higher values therefore indicate fewer depressive symptoms. This direction transformation was also applied to the observed data used for descriptive analyses. Data are presented as mean (SD) using available observations before multiple imputation. Column headings indicate the total number of participants in each group; the number of available observations varies by characteristic. Q1–Q4 denote groups defined by the quartiles of DDS: Q1, 0–3; Q2, >3–5; Q3, >5–6; and Q4, >6–11. BMI, body mass index; DDS, Dietary Diversity Score; MMSE, Mini-Mental State Examination; SD, standard deviation.

**Table 3:** Fit indices of the one-factor IC model across five imputed datasets.

Imputed Dataset	CFI	TLI	RMSEA	SRMR
1	0.9964	0.9929	0.0256	0.0122
2	0.9968	0.9935	0.0243	0.0116
3	0.9974	0.9948	0.0217	0.0107
4	0.9962	0.9925	0.0262	0.0125
5	0.9969	0.9938	0.0237	0.0114

**Table 4:** Associations of the dietary diversity score with intrinsic capacity score and low intrinsic capacity Score.

	Model 1 ^a^	Model 2 ^b^	Model 3 ^c^
**Linear regression**	*β* (95% CI)	*β* (95% CI)	*β* (95% CI)
DDS (per 1-point increase)	0.081 (0.075, 0.087)	0.036 (0.032, 0.041)	0.034 (0.029, 0.039)
**Logistic regression**	OR (95% CI)	OR (95% CI)	OR (95% CI)
DDS quartiles			
Q1	1.00 (Ref)	1.00 (Ref)	1.00 (Ref)
Q2	0.724 (0.666, 0.787)	0.810 (0.722, 0.908)	0.819 (0.729, 0.921)
Q3	0.596 (0.535, 0.664)	0.664 (0.570, 0.773)	0.672 (0.576, 0.784)
Q4	0.407 (0.372, 0.445)	0.560 (0.491, 0.639)	0.566 (0.493, 0.650)
*p* for trend	<0.001	<0.001	<0.001

^a^ Model 1 was unadjusted. ^b^ Model 2 was adjusted for age, sex, residence, education level, income, and marital status. ^c^ Model 3 was further adjusted for smoking, alcohol consumption, sleep duration, regular exercise, hypertension, diabetes, arthritis, and dementia. All models included 15,166 participants. Estimates were pooled across five imputed datasets using Rubin’s rules. Low IC was defined as an IC factor score below the median within each imputed dataset. Q1–Q4 denote DDS quartile groups: Q1, 0–3; Q2, >3–5; Q3, >5–6; and Q4, >6–11. Tests for trend modeled quartile groups as an ordinal variable. CI, confidence interval; DDS, Dietary Diversity Score; IC, intrinsic capacity; OR, odds ratio.

**Table 5:** Associations between DDS and individual intrinsic capacity domains.

IC Domain ^a^	Outcome Indicator	Model	Effect Estimate per 1-Point Increase in DDS ^b^	*p* Value
Cognition	MMSE score ^c^	Linear regression	*β* = 0.305 (95% CI: 0.254, 0.356)	<0.001
Locomotion	Physical function score	Linear regression	*β* = 0.052 (95% CI: 0.039, 0.066)	<0.001
Psychological function	Direction-aligned psychological score ^d^	Linear regression	*β* = 0.050 (95% CI: 0.045, 0.055)	<0.001
Sensory function	Sensory score	Linear regression	*β* = 0.008 (95% CI: 0.006, 0.010)	<0.001
Vitality	BMI within the age-specific reference range ^e^	Logistic regression	OR = 1.024 (95% CI: 1.007, 1.040)	0.004

^a^ IC, intrinsic capacity; ^b^ DDS, dietary diversity score; ^c^ MMSE, Mini-Mental State Examination; ^d^ For the psychological indicator, regression coefficients are expressed in units of item score multiplied by −1; positive coefficients indicate fewer depressive symptoms; ^e^ BMI, body mass index; CI, confidence interval. Linear regression was used for continuous IC domain scores, and logistic regression was used for the binary vitality indicator. Models were adjusted for age, sex, residence, education level, household income, marital status, smoking, alcohol consumption, sleep duration, regular exercise, hypertension, diabetes, arthritis, and dementia. Because the IC domains were measured on different scales, effect estimates are not directly comparable across domains.

## Data Availability

The datasets used in this study are publicly available in the Chinese Longitudinal Healthy Longevity Survey (CLHLS) data archives: https://opendata.pku.edu.cn/dataverse/CHADS (accessed on 2 June 2026).

## Supplementary Materials

The following supporting information can be downloaded at: https://www.mdpi.com/article/10.3390/nu18213568/s1, Table S1. Pre-imputation characteristics of participants included in and excluded from the analytic sample. Table S2. Apparent performance of the fully adjusted logistic regression model across five imputed datasets. Table S3. Primary and sensitivity analyses of the association between dietary diversity and low intrinsic capacity.

## Abbreviations

The following abbreviations are used in this manuscript:

BMI	Body mass index
CES-D	Center for Epidemiologic Studies Depression Scale
CFA	Confirmatory factor analysis
CFI	Comparative fit index
CI	Confidence interval
CLHLS	Chinese Longitudinal Healthy Longevity Survey
DDS	Dietary diversity score
IC	Intrinsic capacity
IQR	Interquartile range
MICE	Multiple imputation by chained equations
MMSE	Mini-Mental State Examination
OR	Odds ratio
Q1	First quartile of dietary diversity score
Q2	Second quartile of dietary diversity score
Q3	Third quartile of dietary diversity score
Q4	Fourth quartile of dietary diversity score
RCS	Restricted cubic spline
Ref	Reference group
RMB	Renminbi (Chinese yuan)
RMSEA	Root mean square error of approximation
SD	Standard deviation
SRMR	Standardized root mean square residual
TLI	Tucker–Lewis index
WHO	World Health Organization
