# Supplementary material for: Association Between Dietary Diversity and Intrinsic Capacity Among Chinese Older Adults: A Cross-Sectional Study Based on the CLHLS 2018
Source: Nutrients. 2026 Oct 28;18(21):3568. doi: 10.3390/nu18213568 (PMC13765379; doi:10.3390/nu18213568)
Supplement: Supplementary file 1 [file nutrients-18-03568-s001.zip › nutrients-4647978-supplementary.pdf]

Supplementary Materials

Table S1. Pre-imputation characteristics of participants included in and excluded from the analytic sample.

Characteristics	Included (n = 15,166)	Excluded (n = 605)	SMD
**Age, years**	85.39 (11.56)	90.80 (11.12)	0.477
Sex			0.182
Male	6,657 (43.9)	212 (35.0)	
Female	8,509 (56.1)	393 (65.0)	
**Residence**			0.113
Urban	2,156 (16.6)	54 (12.6)	
Rural	10,868 (83.4)	376 (87.4)	
**Marital status**			0.449
Married	5,962 (39.7)	103 (20.6)	
Separated	266 (1.8)	6 (1.2)	
Divorced	51 (0.3)	0 (0.0)	
Widowed	8,613 (57.4)	380 (76.2)	
Never married	126 (0.8)	10 (2.0)	
**Education level**			0.369
No formal education	6,515 (50.0)	275 (65.9)	
Primary school	4,088 (31.4)	107 (25.7)	
Middle school or above	2,417 (18.6)	35 (8.4)	
**Household income,RMB**	20,000 [5,000–40,000]	20,000 [6,000–40,000]	0.008
**Smoking status**			0.248
No	12,778 (85.1)	465 (92.8)	
Yes	2,239 (14.9)	36 (7.2)	
**Alcohol consumption**			0.208
No	12,805 (85.7)	451 (92.2)	
Yes	2,129 (14.3)	38 (7.8)	
**Hypertension**			0.054
No	8,023 (56.9)	242 (59.6)	
Yes	6,066 (43.1)	164 (40.4)	
**Diabetes**			0.113
No	12,189 (89.8)	343 (93.0)	
Yes	1,385 (10.2)	26 (7.0)	
**Arthritis**			0.169
No	11,961 (88.4)	332 (93.3)	
Yes	1,569 (11.6)	24 (6.7)	
**Dementia**			0.385
No	13,178 (97.7)	318 (87.8)	
Yes	317 (2.3)	44 (12.2)	
**Sleep Duration, hours**	7.45 (2.40)	8.12 (3.09)	0.242
**Regular Exercise, n(%)**			0.425
No	10410 (69.6)	413 (86.8)	
Yes	4574 (30.4)	63 (13.2)	

Notes: Data are presented as mean (SD), median [25th-75th percentiles], or n (%). Comparisons were restricted to participants aged 65 years or older and used observed data before multiple imputation.

Percentages were calculated using non-missing observations within each group and may not sum to 100% because of rounding. Available sample sizes therefore differed across variables. For categorical variables, a single overall SMD is reported for each variable rather than for individual categories. SMD, standardized mean difference; SD, standard deviation.

Table S2. Apparent performance of the fully adjusted logistic regression model across five imputed datasets.

Imputed dataset	AUC	McFadden's pseudo-R^2
1	0.8804	0.3729
2	0.881	0.374
3	0.8802	0.3721
4	0.8813	0.3748
5	0.8805	0.3729
Mean (range)	0.8807 (0.8802-0.8813)	0.3733 (0.3721-0.3748)

Each model included 15,166 participants. Performance measures were calculated for Model 3 containing DDS11 quartile groups and the full covariate set. Measures were evaluated in the same datasets used for model fitting and therefore represent apparent performance. Means and ranges are descriptive summaries across imputations and were not pooled using Rubin's rules. AUC, area under the receiver operating characteristic curve.

Table S3. Primary and sensitivity analyses of the association between dietary diversity and low intrinsic capacity.

Analysis	N	Quartile comparison	OR (95% CI)	P value
Primary analysis: DDS11 with age-specific BMI definition	15,166	Q2 vs. Q1	0.819 (0.729-0.921)	<0.001
		Q3 vs. Q1	0.672 (0.576-0.784)	<0.001
		Q4 vs. Q1	0.566 (0.493-0.650)	<0.001
IC reconstructed without the BMI-based vitality indicator	15,166	Q2 vs. Q1	0.817 (0.724-0.922)	0.001
		Q3 vs. Q1	0.689 (0.596-0.796)	<0.001
		Q4 vs. Q1	0.574 (0.500-0.658)	<0.001
Low IC defined as the lowest tertile of IC factor scores	15,166	Q2 vs. Q1	0.786 (0.701-0.882)	<0.001
		Q3 vs. Q1	0.682 (0.587-0.793)	<0.001
		Q4 vs. Q1	0.599 (0.519-0.692)	<0.001

Low IC defined as the lowest quartile of IC factor scores	15,166	Q2 vs. Q1	0.764 (0.681-0.858)	<0.001
		Q3 vs. Q1	0.679 (0.577-0.800)	<0.001
		Q4 vs. Q1	0.546 (0.469-0.636)	<0.001
Vitality indicator based on the adult BMI definition	15,166	Q2 vs. Q1	0.813 (0.730-0.906)	<0.001
		Q3 vs. Q1	0.687 (0.596-0.793)	<0.001
		Q4 vs. Q1	0.572 (0.503-0.651)	<0.001
Alternative reverse-coded DDS13	15,166	Q2 vs. Q1	0.784 (0.692-0.888)	<0.001
		Q3 vs. Q1	0.638 (0.559-0.727)	<0.001
		Q4 vs. Q1	0.550 (0.463-0.653)	<0.001
IC reconstructed without the psychological domain	15,166	Q2 vs. Q1	0.816 (0.720-0.925)	0.002
		Q3 vs. Q1	0.709 (0.611-0.822)	<0.001
		Q4 vs. Q1	0.618 (0.539-0.708)	<0.001

All models were adjusted for age, sex, residence, marital status, education level, household income, smoking, alcohol consumption, sleep duration, regular exercise, hypertension, diabetes, arthritis, and dementia. Estimates were pooled across five imputed datasets using Rubin's rules. Q1 was the reference group in each analysis.

In the primary analysis, the BMI-based vitality indicator used age-specific reference ranges of 20.0–26.9 kg/m^2 for participants aged 65–79 years and 22.0–26.9 kg/m^2 for those aged ≥80 years. Low IC was defined as an IC factor score below the median within each imputed dataset, except in analyses using the lowest tertile or lowest quartile. For analyses excluding the BMI-based vitality indicator or the psychological domain or using the adult BMI definition (18.5 ≤ BMI <24.0 kg/m^2), IC factor scores were reconstructed and the median threshold was recalculated within each imputed dataset.

Reverse-coded DDS13 included the 11 primary food groups plus sugar and pickled vegetables, with the latter two items reverse-coded. DDS11 quartile groups were defined as Q1, 0-3; Q2, >3-5; Q3, >5-6; and Q4, >6-11. Reverse-coded DDS13 quartile groups were defined as Q1, 0–4; Q2, >4-6; Q3, >6-8; and Q4, >8-13. The DDS11 and reverse-coded DDS13 analyses included the same participants and retained the primary IC classification and imputed covariates.

BMI, body mass index; CI, confidence interval; DDS, Dietary Diversity Score; IC, intrinsic capacity; OR, odds ratio.
